# Problems and alternatives of testing significance using null hypothesis and *P*-value in food research

**DOI:** 10.1007/s10068-023-01348-4

**Published:** 2023-05-30

**Authors:** Won-Seok Choi

**Affiliations:** Department of Food Science and Technology, Korea National University of Transportation, Jeungpyeong-gun, 27909 Chungbuk Republic of Korea

**Keywords:** Null hypothesis significance test, *P*-value, Confidence interval, Effect size, Bayesian statistics

## Abstract

A testing method to identify statistically significant differences by comparing the significance level and the probability value based on the Null Hypothesis Significance Test (NHST) has been used in food research. However, problems with this testing method have been discussed. Several alternatives to the NHST and the *P*-value test methods have been proposed including lowering the *P*-value threshold and using confidence interval (CI), effect size, and Bayesian statistics. The CI estimates the extent of the effect or difference and determines the presence or absence of statistical significance. The effect size index determines the degree of effect difference and allows for the comparison of various statistical results. Bayesian statistics enable predictions to be made even when only a small amount of data is available. In conclusion, CI, effect size, and Bayesian statistics can complement or replace traditional statistical tests in food research by replacing the use of NHST and *P*-value.

## Introduction

In food of animal or plant resources research, quantitative methods such as experimental research are mainly used. After food research became a separate field of applied science, the use of statistical methods that use the null hypothesis and the *P*-value increased. This was due in part to Fisher, a pioneer in the field of statistics, who mentioned these methods in a tea- tasting sensory test (Fisher, 1935, 1936). In food research, statistical significance at a *P*-value of 0.05 (5%) first appeared in the early 1940s (Eheart and Sholes, 1945; Griswold and Wharton, 1941). Only in the early 1970s, the statistical test procedure was described in the materials and methods of papers (Bouton et al., 1975; Froning et al., 1971; Reddy et al., 1970). In Korea, it was not until the 1990s that statistical tests began to be used in food research papers using animal or plant materials on a variety of topics beyond just sensory tests (Chung et al., 1991; Kim and Lee, 1998; Shin et al., 1991).

The expression “showed a significant difference (at a significance level of 0.05 (5%))” was used to determine whether difference between the control and the experimental groups was statistically significant by comparing the significance level (α) and probability (*P*) based on the Null Hypothesis Significance Test (NHST) (Goodman, 1999; Wasserstein et al., 2019). As previously mentioned, this statistical verification method was started in the early 20th century by Fisher (Bandit and Boen, 1972).

In food research, the significance of the differences is determined using the *P*-value based on the NHST theory when comparing the experimental group of a new method or material to the control group. This statistical reasoning method is a common practice in food research, especially in Korean food research. However, the problems with NHST were actually raised 50 years ago in 1972 (Edwards, 1972). While there has been more recent attention drawn to these problems. In 1988, the International Medical Journal Editors' Committee recognized the problem of using NHST and *P*-value and requested that statistics be reported using confidence interval (CI) instead (Bailar and Mosteller, 1988). Some social science journals even banned using the NHST method in 2015 (Trafimow and Marks, 2015). In addition, the American Statistical Association (ASA) has published a statement on statistical significance and *P-*value, arguing that passing a certain threshold (significance level) the *P*-value should not be the basis for making scientific conclusions, business, or policy decisions (Ronald and Nicole, 2016).

Although there are many reasons for the continued use of NHST and *P*-values in food research despite these controversies, one of the key reasons is that using NHST and *P*-value in statistical testing has become universal and commonplace as a mathematical formula for the solution. In addition, it may be because of the denying the results of numerous published papers or acknowledging that there may be errors by judging whether there is a difference from the control group only with the *P*-value based on the NHST theory. The author is no exception to this conventional behavior and burden. Nevertheless, it is clear that there is a problem in blindly using NHST and *P* values, and there is a need for gradual improvement.

Several methods have been proposed as alternatives to using NHST and *P*-value. However, the most prominent ones include lowering the *P*-value threshold and using confidence interval (CI), effect size, and Bayesian statistics (Benjamin et al., 2018; Joo et al., 1994; Lee, 2013, 2016; Sullivan and Feinn, 2012; Yeo, 2021).

In this paper, I will discuss the problems of using NHST and *P*-value. I will also provide an introduction to basic knowledge and related details on complementary or alternative methods for food researchers who may not have a strong background in statistics. These methods include lowering the *P*-value threshold, and using CI, effect size, and Bayesian statistics. I will argue that using dichotomous statistical tests based solely on NHST and *P*-value should be avoided in food research, and that researchers should gradually incorporate supplementary or alternative statistical testing methods such as CI, effect size, and Bayesian statistics, which can provide more diverse information from the same data.

## Meaning of NHST and *P*-value

The NHST used in statistics is a part of the verification method for inferring the characteristics of the entire population. NHST is used to estimate the characteristics of the population from samples collected from the entire population when it is not possible to obtain information (data) from the entire population, and testing is conducted based on hypotheses such as “no difference” or “no effect” (null hypothesis). The null hypothesis is generally the opposite of the hypothesis the researcher intends to infer (alternative hypothesis), which the researcher wants to reject (Joo et al., 1994; Verdam et al., 2014; Yeo, 2021).

In general, rejection of the null hypothesis in NHST is determined by comparing the significance level and the *P* value. Here, the *P* value refers to the probability value at which the data value of the sample can be obtained under the condition that the null hypothesis is true (Cohen, 2011; Goodman, 1999; Trafimow and Rice, 2009). That is, a *P* value of 0.01 means that the probability of obtaining a sample data value (with a difference or effect greater than or equal to the usual level) from the population for which the null hypothesis is true is a very rare (based on significance level of 0.05) with a probability of 1% (0.01), and a *P*-value of 0.10 is interpreted as the probability of obtaining a sample statistic (sample data value) from the population for which the null hypothesis is true is a relatively high probability (based on significance level of 0.05) with a probability of 10% (0.1). Very high or very rare is a relative concept that requires a criterion and the criterion is called the significance level (generally 0.05 (5%)). If the *P*-value is smaller than the significance level, it is considered that an unlikely event has occurred. As a result, the null hypothesis is rejected and deemed to have statistical significance.

In fact, NHST is a test method that combines Fisher's significance test and Neyman and Pearson's hypothesis test (Lee, 2013). These masters of statistics belonged to different schools, and Fisher, the founder of the concept of the null hypothesis, used the *P*-value only to reject the null hypothesis, and set the rejection criterion (significance level) at a “convenient and flexible” value of 0.05. On the other hand, Neyman and Pearson supplemented Fisher’s theory and formulated the concept of alternative hypotheses in order to draw mathematical and scientific conclusions, providing a “fixed” criterion for the selection (adoption of the null hypothesis) and rejection (adoption of the alternative hypothesis) of the hypothesis with the use of Type I error (false rejection) probability as α. In other words, if Fisher’s test recommended flexible conclusions, Neyman and Pearson insisted on making strict conclusions. The problem arose when a new theory emerged amid the confrontation and debate between the two schools of thought. Fisher’s *P* value began to be used with Neyman and Pearson's α, which corresponded to Fisher’s significance level to draw a mathematically rigorous conclusion (Perezgonzalez, 2015). Many current problems are the result of blindly using this testing method, which is a simple blend of different schools of thought in an attempt to unify theories.

## Problems with using NHST and *P*-values

As mentioned in the introduction, the problems of making statistical inference using NHST and *P*-value are as follows (Lee, 2013; Sullivan and Feinn, 2012; Yeo, 2021).

First of all, the most problematic of misunderstandings about the *P*-value is that it misinterprets “the probability of observing the data value of the sample under the condition that the null hypothesis is correct (true)” as “the probability of the null hypothesis being true based on the observed sample statistic” (Carver, 1978; Nickerson, 2000). Since the value of *P* is calculated on the premise that the null hypothesis is true, it cannot be a value of the probability that the null hypothesis is true. A *P*-value of 0.01 means that the probability of obtaining a sample data value is 1% (0.01) from the population for which the null hypothesis is true, which is a very rare (based on significance level of 0.05) probability and does not mean that there is only a 1% chance that the null hypothesis is true. In other words, when the null hypothesis is rejected, the interpretation that the probability of this being an error is 1% (a very rare probability based on the significance level of 0.05) is an incorrect interpretation. In general, it is wrong to think of the *P*-value as the probability of making an error, i.e., the error rate, and Sellke et al. (2001) reported that for a *P* value of 0.05, the error rate is estimated as “at least 29% (typically around 50%).”

Second, the NHST lacks an explanation of the results, and may sometimes provide no meaning at all. When establishing a null hypothesis such as “there is no (performance) effect of the program” or “there is no difference between the two groups” in a study, the researcher might be interested in the extent of the effect, i.e., effect size along with the presence or absence of an effect, if indeed there was one. When the null hypothesis is rejected, the effect is “statistically” significant, but it is not known whether this effect is “actually” meaningful or meaningless. The same is true for the analysis of differences between the two groups. In the natural sciences, especially in physics, theoretically, the effect can have a value of 0 (zero), but in reality, the case where the effect of a program is 0 is extremely rare; particularly, in the field of social science, it is almost impossible for an effect to have a value of zero. Between the two groups, it is highly probable that there will be a difference of 0 or more mostly due to the surrounding conditions other than the essence, such as the difference in the test method and the number of samples in the groups (McShane and Gal, 2017; Yeo, 2021).

Third, the *P*-value is inevitably affected by the number of samples (cases). The larger the number of samples, the smaller the *P*-value, and conversely, the smaller the number of samples, the higher the *P*-value. Therefore, if the researcher intends to show a significant effect or difference by lowering the *P*-value, the researcher has a higher probability of probabilistic success if the number of samples is increased, which leads to the misunderstanding that the smaller the *P*-value, the greater the effect. That is, the usefulness of the *P*-value decreases as the number of samples increases (Sullivan and Feinn, 2012).

Fourth, papers that performed statistical inference using NHST and *P*-value have no choice but to interpret the results dichotomously. Determining significance by the size of the *P*-value at a specific statistical significance level (generally 0.05) has the advantage of being simple and clear to judge the result, but can it be said that the effect is 0 or there is no difference if the *P*-value is 0.051 at the significance level of 0.05? In other words, can we say that the 0.002 difference between the *P*-values 0.051 and 0.049 is the absolute difference that will decide the fate? Also, the significance level of 0.05 was an arbitrary and reference only value suggested by Fisher, but now many researchers regard it as an almost absolute reference point (McShane and Gal, 2017).

Fifth, it leads to publication bias. Publication bias refers to the selection and publication of only papers with favorable results by an academic organization (Gerber and Malhotra, 2008). The use of NHST and *P*-value is likely to categorize various studies dichotomously into successful and failed studies, leading to the publication of only the studies with so-called statistical significance showing a *P*-value that is lower than the significance level (Bruns and Ioannidis, 2016; Fanelli, 2012). This could further lead to side effects such as over-interpretation of study results or preventing various results from being published (Simonsohn et al., 2014).

Sixth, NHST and *P*-value are not correlated with the reproducibility of results, which refers to the ability to obtain the same or similar results when published studies are repeatedly performed in the same way (Plucker and Makel, 2021). The majority of readers believe that papers that use NHST and *P*-value to publish statistically significant results have reproducibility. However, unfortunately, according to the study by Ioannidis (Ioannidis, 2005), although there are differences between studies, a significant number of papers published in the medical field did not reproduce the statistical significance. This paper is currently cited more than 7000 times (PLOS MEDICINE, 2023). Even when the *P* value of the research results is 0.001, the reproducibility of the research results cannot be guaranteed (Yeo, 2021).

Meanwhile, the American Statistical Association (ASA) mentioned the following principles regarding *P*-values (Ronald and Nicole, 2016).

1. The *P*-value indicates how much the data is inconsistent with a specific statistical model (hypothesis) rather than interpreting it as a probability that a given hypothesis is true based on the sample dataset. In other words, if the basic assumption used to calculate the *P*-value is maintained, the smaller the *P*-value, the greater the statistical discrepancy between the null hypothesis and the data;
2. The *P*-value does not measure the probability that the research hypothesis is true or the probability that the data were created by chance;
3. One should not make any scientific conclusion, business or policy decisions based only on the passing of a specific threshold (significance level) of the *P*-value;
4. For proper inference, it is necessary to report all statistics transparently and completely in addition to the *P*-value;
5. The *P*-value or statistical significance does not measure the size of the effect or the importance of the outcome;
6. The *P*-value by itself is not a good evidence for a statistical model or hypothesis (e.g., whether the null hypothesis is true or false).

As an alternative to the problem of using NHST and *P*-value, several researchers have proposed lowering the *P*-value threshold, and using CIs, effect sizes, and Bayesian statistics (Benjamin et al., 2018; Joo et al., 1994; Lee, 2013, 2016; Lin et al., 2013).

## Lowering the *P*-value threshold

Benjamin et al. (2018) numerically analyzed the problems that occur when using a *P*-value of 0.05. As a result, when the two-sided *P*-value is 0.05, the minimum probability that the alternative (research) hypothesis is true is 75%, which means that the maximum probability that the null hypothesis is also true is also 25%. In order to solve this problem, they argued that the significance level should be lowered and the *P*-value should be 0.005. According to the simulation results of these researchers, when the *P*-value is 0.005, the probability that the alternative (research) hypothesis is true increased about 6.8 times compared to when the *P*-value was 0.05, and the percentage of false positives decreased by about 6.6 times (33% → 5%). In addition, it was argued that the study results with a *P*-value of 0.05 to 0.005 are suggestive results, which are inadequate and should be treated as a study that requires further research. Opposition to this argument has also been published. Ioannidis (2018) argued that lowering the threshold of *P*-value to 0.005 is a temporary measure, a lower threshold may be preferred, and the magnitude of the effect may be more exaggerated than in the previous case. The power (1-β) corresponding to a *P*-value of 0.0001 was reported to be about 0.85 (sample size 100) (Norman, 2019).

## Confidence interval

When estimating population parameters from study data (sample), the best estimate is a single value, which is called point estimation. This is one of the infinitely possible numbers, and the probability of being able to estimate the single value is very low. Therefore, it is more preferable to present the estimate as a value of a range (interval), and when the expected values of an unknown parameter, consistent with the observed data (within a limited range), are expressed as a series of ranges, it is called a CI and this estimation is called interval estimation (Langman, 1986). The range of the CI is affected by the number of variables and the confidence level, which is an arbitrary number (Lee, 2016; So, 2016). Confidence level of 95% is mainly used, and it is directly related to the significance level, so it corresponds to a value of (1–significance level) × 100. That is, the 95% CI corresponds to a significance level of 0.05. In interpreting this, if the 95% confidence level interval for the population mean difference or population ratio between the two groups does not include the null value (0 or 1, respectively) of the parameter and is included in the 99% CI, the *P*-value of this parameter can be said to be greater than 0.01 and less than 0.05. That is, it is not statistically significant at the significance level of 0.01, but is at the significance level of 0.05. However, the interpretation of this requires attention as in the interpretation of the *P* value. In calculating the 95% CI of the mean value from the sample, it should be interpreted as meaning that the population mean value is included in 95 CI**s** (that is, 95% CI) among the CIs calculated from 100 samples obtained by the same method from the same population; it would be erroneous to interpret this as a 95% probability (or the population mean is included with 95% probability) that the 95% CI of the mean value calculated from a “single sample” contains the population mean value.

If two groups with a small sample size satisfy the assumption of normal distribution and equal variance, the CI for the difference in mean values between the two groups can be obtained from the following equation using t-statistics (Lee, 2016).

$$ S_{pooled}^{2} = \sqrt {\frac{{\left( {n_{1} - 1} \right)s_{1}^{2} + \left( {n_{2} - 1} \right)s_{2}^{2} }}{{n_{1} + n_{2} - 2}}} $$

(${s}_{pooled}$, n_i_; pooled sample variance at equal variance assumption, sample size of group i) 

Confidence interval;

$$ \left[ {({\overline{\text{x}}}_{1} - {\overline{\text{x}}}_{2} ) - t_{\alpha }^{d} f \times S_{pooled} \sqrt {\frac{1}{{n_{1} }} + \frac{1}{{n_{2} }}} , ({\overline{\text{x}}}_{1} - {\overline{\text{x}}}_{2} ) + t_{\alpha }^{d} f \times S_{pooled} \sqrt {\frac{1}{{n_{1} }} + \frac{1}{{n_{2} }}} } \right] $$

($\overline{Xi}$, n_i_, ${t}_{\alpha }^{df}$; mean of group i, sample size of group i, critical value of the t-distribution for the probability α and degrees of freedom)

I would like to explain the difference between using NHST and *P*-value and using CIs. When comparing the effects of the existing treatment A and the new treatment B, if the NHST-based *P* value is 0.06, it is generally concluded that “there is no significant difference between the two treatments”. In this case, when the *P*-value is expressed as *P* = 0.06 rather than *P* > 0.05, a more diverse meaning can be provided. Nevertheless, the *P*-value only judges the significance of the difference in the treatment effect between the two treatments, but does not explain the extent of the difference in the treatment effect (Joo et al., 1994).

**Fig. 1:**
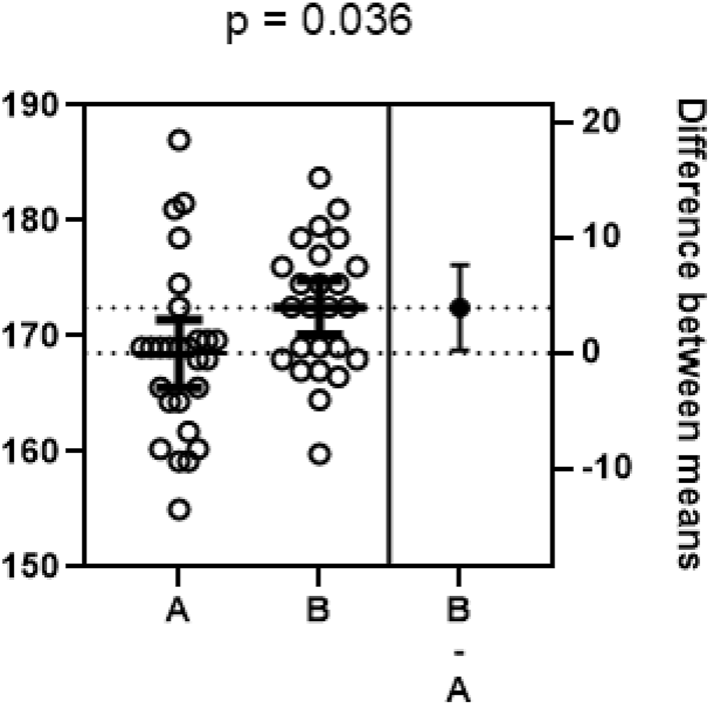
95% confidence intervals. Adapted from Choi and Han (2017)

In Fig. 1. the individual 95% CIs for the study results of A and B and the 95% CI for the difference between the population means of the two studies was shown (Choi and Han, 2017). It can be thought that there is no significant difference between A and B at the 0.05 (5%) significance level because the individual CIs of A and B partially overlap, but this would be an incorrect interpretation; given the 95% CI (far right, B-A) for the population mean value difference between A and B, the correct interpretation would be that there is a statistically significant difference at the significance level of 0.05 because 0 (null value for the mean value difference) is not included. Also, since each CI represents the expected size of the effect, which means that it can have an effect within the range, it can be estimated that B is more effective than A (Choi and Han, 2017; Joo et al., 1994; Lee, 2016). In other words, the CI provides information on the extent (size) as well as the statistical significance of the effect or difference. It is possible to compare more than 3 groups by this method.

Usually, the distribution of numerical values in the CI is not uniformly distributed but roughly follows a normal distribution, so it can be estimated that the data are mainly located in the center rather than at the boundary, which is both ends of the CI. That is, the boundary values of the CI mean that the frequency of the data is small and does not significantly affect the interpretation. Different interpretations may occur depending on the location of the boundary value, but this is not a big problem because such cases are rare. That is, comparing the *P* value with the significance level (e.g., 0.05) is equivalent to focusing all on the boundary value of the CI while ignoring information on the extent (size) of the effect or difference (Joo et al., 1994).

As a result of using CIs to re-interpret 71 clinical trial papers published with the conclusion that there was no effect based on the NHST and P value, Freiman et al. (1978) reported that the results of many studies were misinterpreted and that there were actually effects.

## Effect size

CIs provide some information about the magnitude of effects that NHST and *P* value do not, but they only provide a range of possibilities.

Effect size refers to the difference in standardized scores between groups, i.e., the size of the difference when testing for differences between groups. For example, if the test scores in each subject increased by an average of 30 points (based on 100 points) through a series of programs, the absolute effect size would be 30 points or 30%. Absolute effect sizes are useful when variables are numerical values that have intrinsic meaning (such as sleep time) while the calculated effect size, such as Cohen's d, is used for numerical values that have no intrinsic meaning, such as the Likert scale (Sullivan and Feinn, 2012). The calculated effect size is commonly used in meta-analysis as it enables the comparison of statistical results by solving the difficulty of comparison due to inconsistency in measurement units or using other measurement methods (Kim, 2011; Lee, 2016).

The process of obtaining the effect size index calculated to confirm the difference in the effect between two independent groups (control group and experimental group, etc.) is simple and has the advantage of leading to a more direct interpretation of results. Several types of effect size indices exist, and are mentioned in Table 1 (Lee, 2016; Sullivan and Feinn, 2012).

**Table 1:** Effect size indices

Index	Description	Effect size
*Between group*		
Cohen’s d	d = $\frac{\mathrm{M}1-\mathrm{M}2}{S}$	Small; 0.2
	M_1_-M_2_; difference between the group means (M), *S*; standard deviation of pooled group	Medium; 0.5
		Large; 0.8
		Very large; 1.3
Odd ratio	$\frac{\mathrm{Group }1\mathrm{ odds \, of \, outcome}}{\mathrm{Group }2\mathrm{ odds \, of \, outcome}}$	Small; 1.5
		Medium; 2
		Large; 3
Relative risk or risk ratio	$\frac{\mathrm{Probability of group }1\mathrm{ outcome}}{\mathrm{Probability of group }2\mathrm{ outcome}}$	Small; 2
		Medium; 3
		Large; 4
*Measures of association*		
Pearson’s r correlation	− 1∼1	Small; ± 0.2
		Medium; ± 0.5
		Large; ± 0.8
R^2^ coefficient of determination	0∼1	Small; 0.04
		Medium; 0.25
		Large; 0.64

Adapted from Sullivan and Feinn (2012)

The formula for calculating the d value, which is a representative effect size index, is as follows.

$$ {\text{d}}\, = \,\frac{{({\overline{\text{x}}}_{1} - {\overline{\text{x}}}_{2} )}}{{S_{pooled} }} $$

$$ S_{pooled} = \sqrt {\frac{{\left( {S_{1}^{2} + S_{2}^{2} } \right)}}{2}} $$

($\overline{{X}_{i}},$ ${s}_{pooled}$; mean of group I, pooled sample variance at equal variance assumption) 

According to the criteria proposed by Cohen (1988), a d value of 0.2 is considered a small effect size, a value of 0.5 is considered a medium effect size, and a value of 0.8 is considered a large effect size; however, just as significance level criterion *P* value of 0.05 is a value proposed by Fisher for reference, this value should not be interpreted as an absolute value. Cohen's d = 0.5 means that the mean score of the experimental group is 0.5 SD (standard deviation) higher than the mean score of the control group, which means that about 69% of the control group (standard normal cumulative distribution function value 0.5) is lower than the mean score of the experimental group (Kim, 2015).

A typical example of using an effect size is the following. When aspirin was administered to prevent myocardial infarction to 22,000 subjects for 5 years, a statistically significant result of *P* < 0.00001 was found. Based on this, aspirin was recommended for the prevention of myocardial infarction. However, the effect size of this study was very small, and further studies showed much smaller effects, so the recommendation for aspirin use was revised (Bartolucci et al., 2011). In addition, using the effect size can save us from the dichotomous conclusion resulting from the use of NHST and *P* value; it is also independent of the sample size (Sullivan and Feinn, 2012).

In the paper titled Concept and application of effect size, Kim (2011) asserted that attention should be paid not only to statistical significance but also to practical effect (usefulness), as the calculated effect size, Cohen’s d, ranged between 0.40 and 0.72 for the studies that did not have statistically significant results because the *P* value slightly deviated from 0.05 (0.055 to 0.066).

Despite these various advantages, there are criticisms that another effect not related to the effect size can be ignored by oversimplifying the interpretation of Cohen's d. For example, if there is a drug that is inexpensive and safe, although the effect size is small for curing the patient's disease, the effect size for the patient may be small in the medical aspect, but the effect in terms of economic and social aspect is not so small (McGough and Faraone, 2009).

## Bayesian statistics

Whereas the aforementioned frequentist statistics of Fisher and Neyman–Pearson are a method of estimating fixed-value parameters based on the statistical distribution of a repeated sample, Bayesian statistics is a method of estimating parameters based on the posterior probability that combines the priori probability of parameters with new data (sample) after assuming that the parameter has a probability distribution (prior probability). Therefore, sequential inference is possible, and it is similar to the human thought-judgment process. In other words, the strength of Bayesian statistics is that the previous research data (prior probability) and new research data are placed on a continuous flow line without distinction, therefore are updated (posterior probability) in response to new data in real time; as the data accumulates, the estimation becomes more accurate. In addition, Bayesian statistics have the advantage of being able to make estimates in various environments as there is no null or alternative hypotheses; they provide reliable results even when the sample size is small, do not require a significance level, and do not depend on the limits of assumption of multivariate normality and assumption of equal variance. On the other hand, since prior probabilities are used, the subjectivity of the researcher may be involved and there is the disadvantage of using very complex mathematics; however, these shortcomings can be overcome by using uninformative prior probability or by the development of computer calculation functions (Kim et al., 2016; Lee, 2013; Noh et al., 2014).

By overcoming these limitations, Bayesian statistics are being used in various fields such as IT business for estimating the behavior or search form of Internet consumers, imaging for noise removal, diagnosis through prediction in the medical field, analysis and prediction of surveys and agricultural production, and climate factor analysis. In addition, as the basic study of deep learning model, it is gaining popularity (Kim et al., 2016; Nurminen and Mutanen, 1987; Wang and Campbell, 2013).

Nevertheless, Bayesian statistics is a completely different concept from the existing frequentist statistics, and it is not easy to understand and utilize it for researchers in the food field who do not have a deep academic depth in the statistics.

A typical example of using Bayesian statistics is a case related to COVID-19. It involves calculating the probability of a person who tested positive on a diagnostic kit actually being infected with the disease. The core of the kit is sensitivity and specificity, where sensitivity is “the probability that the diagnostic kit will determine that an infected person has the infection (positive)”, and specificity is “the probability that a person who is not infected will be determined as not infected (negative)”.

Assuming a prevalence rate of (the number of patients with a specific disease/total population) 50% for COVID-19, and sensitivity of 99% and specificity of 90% for the test kit, the probability of actually having the disease when the test result is positive is calculated as follows (Lee, 2013).

$$ {\text{P}}\left( {{\text{D}}\left| {{\text{positive}}} \right.} \right)\, = \,\left[ {{\text{P}}\left( {{\text{positive}}\left| {\text{D}} \right.} \right)\, \times \,{\text{P}}\left( {\text{D}} \right)} \right]/{\text{P}}({\text{positive}}) $$

Here, P(disease│positive) is the probability of having the disease, given a positive test, (corresponds to the “posterior probability” of the disease), P(positive│disease) is sensitivity (“referred to as likelihood”), P(disease) is the probability of having the disease (corresponds to the “prior probability” of the disease, referred to as prevalence), P(positive) is the probability of testing positive, (“marginal probability”). This expression is Bayes’ theorem in Bayesian statistics.

P(positive) can be expressed as the sum of P(positive│D) × P(D), i.e., the probability of true positive and P(positive│not D) × P(not D), i.e., the probability of false positive.

$$ {\text{P}}\left( {{\text{positive}}} \right)\, = \,\left[ {{\text{P}}\left( {{\text{positive}}\left| {\text{D}} \right.} \right)\, \times \,{\text{P}}\left( {\text{D}} \right)} \right]\, + \,[{\text{P}}\left( {{\text{positive}}\left| {\left| {\text{not D}} \right.} \right.} \right)\, \times \,{\text{P}}\left( {\text{not D}} \right)] $$

Here, P(positive│not D) is the probability that a healthy person may test positive, referred to as false positive, and is calculated as ‘1- specificity’; P(not D) is the probability of being healthy and is calculated as ‘1-prevalence’.

If the test result came out to be positive using this formula, P(D│positive) = 0.99 × 0.5/((0.99 × 0.5) + (0.1 × 0.5)) = 0.908, i.e., 90.8% is the probability of actually having COVID-19.

Unlike the calculated value of 90.8%, many may think that the probability of actually having the disease to be 99%, but this is the result of confusing the sensitivity of the kit (P(positive│D)) with the probability of actually having the disease (P(D│positive)) when test result is positive. The posterior probability, P(D│positive) is directly and decisively influenced by the prior probability, P(D), so if it is assumed that the prevalence is low (P(disease) = 10%), the probability of actually having the disease may drop significantly (52.4%) even when the positive result is obtained using the diagnostic kit having the same sensitivity (99%) and specificity (90%).

In Bayesian statistics, various statistical analysis programs such as SPSS and JAMOVI can still be used to perform common statistical analyses like correlation, regression, and ANOVA.

Bayesian statistics can express both subjective concepts, such as “approximately,” and objective concepts, such as “numerical values,” which can be advantageous for statistical estimation in fields related to food, such as sensory science, that encompass many social scientific aspects.

In recent years, food research has increasingly utilized large datasets. Bayesian statistics, as a fusion of artificial intelligence and statistics, is one of the techniques that can solve the problem of time-consuming and difficult to manage classical (frequentist) statistical analysis. Due to its advantages, Bayesian statistics is widely used in a variety of fields. However, it has only recently made its presence felt in the food industry (Van Boekel, 2004).

Initially, modeling with Bayesian statistics was primarily focused on microbial risk assessment in food production, using structured and simple models that added new information in an organized manner (Barker et al., 2002). Bayesian statistics is also used in kinetics to understand food reactions and influence product and process design. Bayesian statistics has the benefit of being easy to interpret and can be applied to a wide variety of complex models with varying degrees of complexity (Van Boekel, 2020).

Bayesian statistics can be applied in various ways to the food industry. It can estimate the shelf life of food products by analyzing data on degradation and considering factors such as temperature, humidity, and pH (Calle et al., 2006; Luong et al., 2022). In sensory analysis, Bayesian statistics can evaluate food samples and estimate the distribution of sensory attributes in the population, helping food companies make informed decisions about product development (Calle et al., 2006). Bayesian statistics is also useful for assessing the risk of foodborne illness from microbial contamination (Oishi et al., 2021). By incorporating uncertainty, Bayesian statistics can provide more accurate estimates and predictions in all these applications. Overall, Bayesian statistics is a powerful tool for food scientists to draw more reliable conclusions and make more accurate predictions in their studies.

To summarize, the contents are as follows. Although the statistical testing method of determining statistically significant differences by comparing significance level (α) and probability (P) based on the NHST has been commonly used in food research, problems with this testing method have been identified, and some social science journals even banned the use of the NHST method in 2015. Given the limitations of NHST and *P*-value, several alternative methods have been proposed, including lowering the *P*-value threshold and utilizing measures such as CI, effect size, and Bayesian statistics. Lowering the threshold for *P*-value to 0.005, increases the probability that the alternative hypothesis is true by about 6.8 times compared to when the *P*-value is 0.05, while reducing the ratio of false positives by approximately 6.6 times (33% → 5%). The CI is another alternative, that provides information about the extent of effect or difference and the presence or absence of statistical significance unlike the *P*-value, which only determines the statistical significance of an effect or difference. The third alternative is the use of effect size. Using an effect size index such as Cohen's d can distinguish the extent of effect difference and enables the comparison of various statistical results by solving the difficulty in comparison due to the inconsistency of measurement units and the use of different measurement methods. Lastly, Bayesian statistics can be used. Bayesian statistics can express the subjective concept of “approximately” as an objective concept of “numerical value”, which can be useful for statistical estimation in the food field, such as sensory science, which involves many social scientific aspects. Additionally, Bayesian statistics can be updated in real time by incorporating new data into the previous research data (prior probability), resulting in increasingly accurate as more data is accumulated.

In conclusion, dichotomous statistical analysis using NHST and *P*-values in food research is problematic. As a complement or alternative, it is worth considering a gradual transition to using relatively simple statistical concepts such as CI and effect size, which can provide more information about your results compared to dichotomous statistical analysis using NHST and *P*-values. Additionally, although Bayesian statistics are more complex, it can also be a valuable alternative to consider.
